# Working Differently, Performing Similarly: Systems Intelligence and Job Crafting as Predictors of Job Performance in a Three-Wave Longitudinal Study

**DOI:** 10.3390/bs15091255

**Published:** 2025-09-14

**Authors:** Sidra Liaquat, Jordi Escartín, Jacqueline Coyle-Shapiro

**Affiliations:** 1 Department of Social and Quantitative Psychology, Universitat de Barcelona, Passeig de la Vall d’Hebron, 171, 08035 Barcelona, Spain; jordiescartin@ub.edu; 2 Institute of Psychiatry, Psychology & Neuroscience, King’s College London, De Crespigny Park, London SE5 8AF, UK; 3 Department of Management, The London School of Economics and Political Science, Houghton Street, London WC2A 2AE, UK; j.a.coyle-shapiro@lse.ac.uk

**Keywords:** systems intelligence, job performance, crafting behaviours, job resources, personal resources, longitudinal analysis, job demands-resources model, resource substitution

## Abstract

In light of a Volatile, Uncertain, Complex and Ambiguous (VUCA) world, the need for employee adaptability is a critical capacity to navigate challenges and facilitate employees thriving in organizations. One important capacity, systems intelligence, captures employees’ ability to think, adapt and act effectively in interactions with systems. In a three-wave longitudinal study, we examine the relationship between systems intelligence (SI), job crafting (JC), and job performance (JP) over time. We employ the job demands-resources model to demonstrate that SI increases JP, hypothesizing that job resources, as manifested in JC, act as mediator between personal resources (SI) and JP. Data were collected from employees in Pakistan working across the banking, telecommunications, information technology, and engineering sectors. In the first wave, 303 participants completed the survey using validated self-report measures, followed by 212 in the second wave, and 99 in the third wave, each two months apart. Our findings show that systems intelligence at Time 1 was positively related to job performance at Time 3 but not Time 2. We found no significant association of SI at Time 1 with JC at Time 2 or Time 3. JC at Time 2 did not mediate the effects of SI at Time 1 on JP at Time 3. However, JC (T1 & T2) had a significant positive effect on JP (T2 & T3). Overall, our findings suggest that the pathways from systems intelligence and job crafting to job performance are independent. This dual pathway to performance has important theoretical implications as well as practical implications for organizations. Organizations can improve team and individual productivity by fostering systems intelligence and promoting job crafting behaviours. This research directs the attention of leaders and HR functions to the value of tailored interventions in developing these abilities and achieving long-term success and adaptive performance in the workforce.

## 1. Introduction

Employees nowadays face multiple workplace challenges, including rapid digital transformation that requires resilience and systems thinking, high-stakes expectations under resource constraints (Fröhlich et al., 2025), and complex interactions that require adaptive problem-solving and emotional intelligence (Drigas et al., 2023). To remain competitive, organizations increasingly demand these adaptive capabilities from their workforce. The concept of systems intelligence (SI) captures these abilities by integrating cognitive, emotional, and systemic capacities that enable individuals to navigate and thrive in complex environments. An individual’s SI level reflects their ability to think, adapt, and act effectively by responding to the basic features of their work context. SI has been derived from systems science (Hamalainen et al., 2018) and the Fifth Discipline (Senge, 1990). Saarinen and Hämäläinen (2004) described SI as “one’s perception of the whole, recognising the reciprocal link of self and systems, which leads to survival behaviours. It is a competence that can be improved by learning” (p. 60). SI comprises eight competencies: systemic perception, positive engagement, wise action, reflection, attunement, involvement in the system, a positive attitude, and effective responsiveness (Törmänen et al., 2016). Systems perception refers to understanding and recognizing systems; attunement refers to the capacity to connect with and engage within systems; reflection is the ability to analyse and think critically about one’s own thought processes. Positive engagement refers to involving meaningful and interactive communication. Spirited discovery is characterized by enthusiasm for exploring innovative ideas. Effective responsiveness refers to timely and appropriate actions. Wise action involves understanding and managing situations with a long-term perspective, whereas positive attitude refers to an overarching optimistic approach towards life.

SI is considered intelligent behaviour and a learned skill that can enhance personal growth, improve individual capabilities, and improve team and organisational behaviours (Hamalainen et al., 2018). People and systems are interconnected because individuals within a system interact according to their mindset and receive feedback from the system. SI has been used across many fields, such as knowledge management, personal development, as well as complex cognitive processes such as designing (Jumisko-Pyykko et al., 2022) and engineering (Hamalainen et al., 2018), showcasing its wide-ranging impact on problem-solving and innovation. Systems intelligence (SI) has been associated with job performance in a peer-evaluation context (Jumisko-Pyykko et al., 2022; Törmänen et al., 2022). We aim to study how SI enables individuals to navigate complex systems and to respond effectively to workplace demands. This capability aligns with the job demands-resources (JD-R) framework. This framework is well adapted to the current study, which examines job crafting with regard to system intelligence, since they elucidate both the motivational processes and the compensatory mechanisms that can protect against resource deficits. Integration of systems intelligence into JD-R adds a novel layer by emphasizing systemic awareness and adaptability to work effectively and dealing with workplace demands.

Job resources refer to the tangible, interpersonal, emotional and structural elements of a job (Demerouti et al., 2001). Job resources serve to facilitate work-related objectives, minimise work-related stressors and their consequent physical and mental effects, and promote individuals’ progression and advancement. In contrast, personal resources are defined as individuals’ inner strengths, such as optimistic self-perceptions associated with resilience and individuals’ beliefs about the ability to effectively manage and shape their environments (Hobfoll et al., 2003; Hobfoll et al., 2018). Therefore, personal resources facilitate goal attainment, alleviate physical and emotional burdens, and foster self-improvement and advancement. Some examples of personal resources include personal skills, self-efficacy, personal effectiveness, optimism, hope, resilience and self-esteem (Xanthopoulou et al., 2007, 2009a).

Empirical evidence supports the positive relationship between job resources (such as job crafting) and performance outcomes (Demerouti et al., 2015; E. Robledo et al., 2019; Rofcanin et al., 2019; Tims et al., 2012). Crafting behaviours have been theorised as a job resource that employees can utilise to optimise their work experience (Bakker & Demerouti, 2007). Employees may proactively change their tasks, relationships or cognitions to create a better fit between their preferences, identities and their job (Lu et al., 2014), and between their job demands and personal strengths (Wrzesniewski & Dutton, 2001). Such changes enhance employees’ sense of control, autonomy, and meaningfulness at work, leading to improved well-being and job performance (Tims et al., 2013, 2015; Neuber et al., 2021). Prior research has demonstrated that employees with adequate personal resources can strengthen their job resources (Bakker & Demerouti, 2014), exhibit confidence, have optimism or create conducive environments for goal achievement (Xanthopoulou et al., 2007). Job resources can also amplify the positive effects of personal resources on job performance, such that when individuals have higher levels of job resources, the positive relationship between personal resources and job performance is stronger (Bakker & Demerouti, 2007; Fröhlich et al., 2025). In addition, (Xanthopoulou et al., 2007; Tang et al., 2024) found that personal resources were positively related to job resources; the latter partially mediated the relationship between personal resources and work outcomes. The JD-R model suggests that both personal resources and job resources are important predictors of job performance. Furthermore, the relationship between personal resources and job performance may be mediated and amplified by job resources. (Bakker & Demerouti, 2014, 2017; Miraglia et al., 2017; D. Robledo et al., 2021).

However, the interplay between personal resources and job resources and their impact on employees’ job performance is unclear. Some studies (Bakker & Demerouti, 2017; Xanthopoulou et al., 2009b) suggest a positive correlation between personal and job resources, others yield mixed or inconclusive results (Schaufeli & Taris, 2014; Galanakis & Tsitouri, 2022). In light of the mixed empirical findings, researchers have called for more longitudinal studies focusing on SI (Jumisko-Pyykko et al., 2022) and the JD-R model (Gonzalez-Mulé et al., 2021) to shed light on the role of personal characteristics (personal resources) as a determinant of job performance and the interplay of job resources between personal resources and job performance. Gonzalez-Mulé et al. (2021) noted that while the JD-R model recognizes the role of personal resources, it does not offer a clear method for incorporating them into its framework. These resources can act as antecedents, mediators, moderators, or a combination of these factors within the model. Despite these interactions, the recent extensions of the JD–R model propose that when multiple resources are simultaneously available, their individual effects may weaken, because they compensate for one another in sustaining work outcomes and well-being (Demerouti & Bakker, 2023). Substitution hypothesis suggests that resources can reinforce or substitute for one another in managing demands (Ross & Mirowsky, 2010; Koltai & Schieman, 2015). Thus, employees with strong personal resources rely less on job resources for performance, whereas those with fewer personal resources depend more heavily on them for well-being and effectiveness.

To begin to address this gap, we aim to advance understanding of the role of personal resources and job resources in explaining work performance using a cross lagged methodology. This study seeks to explore resource substitution theory (Demerouti & Bakker, 2023) in the context of personal resources, job resources and job performance. In doing so, we clarify how different resources interact to influence employee effectiveness. Specifically, we aim to establish whether high personal resources (SI) reduce employees’ dependence on job resources (JC) to achieve performance.

Our study contributes to the existing literature by investigating how systems intelligence (SI) as a personal resource predicts job performance (JP). We hypothesize that personal resources such as SI will predict JP and also positively affect job resources like job crafting. We propose that job crafting will mediate the relationship between SI and job performance. The relationship between SI and JP is crucial because SI enables individuals to cope more successfully with interpersonal interactions, manage conflicts, and cooperate in a more functional way. An examination of this relationship is important as SI has the potential to further facilitate other beneficial job resources, such as job crafting, enabling individuals to modify their work environment to make it more supportive of their strengths and needs. More specifically, the present study aims to investigate the longitudinal relationship between SI, JC, and job performance. In doing so, this study provides insights into the mechanisms that enable employees to perform their jobs more effectively and adapt positively, benefiting not only themselves but also the organizations they work for.

### 1.1. Literature Review and Hypothesis Development

#### 1.1.1. Systems Intelligence and Job Performance

The JD-R model was first introduced by Demerouti et al. (2001) as a framework to explain burnout and engagement. Over time, Bakker and Demerouti (2007, 2017) extended it into a broader motivational framework, applicable across occupations and contexts, emphasizing dual processes: (1) health impairment (high demands drain energy), and (2) motivational (resources foster engagement). According to the JD-R model, personal resources are considered to have an impact on work outcomes, such as job performance. Some researchers (Bakker & Demerouti, 2017; Xanthopoulou et al., 2007, 2009a) argue that personal resources have the potential to boost employees’ resilience and perceived competence. By facilitating the effective management of their surroundings, personal resources can aid in attaining favourable outcomes. As a personal resource, SI is focused on identifying the factors that contribute to human success while working within complex systems. These factors include systemic perception, attunement, positive engagement and effective responsiveness, all of which emphasise the importance of considering the context as a crucial element in achieving successful outcomes (Törmänen et al., 2016).

Previous cross-sectional research has shown a positive link between SI and job performance. Peer evaluated SI was positively correlated with job performance, particularly in the information technology and technical sectors (Hamalainen et al., 2018). Other empirical research also found a positive correlation between overall peer evaluated SI and perceived performance among managers, regardless of gender, age, and organizational size (Törmänen et al., 2021), organizational-based SI and high performance (Jumisko-Pyykko et al., 2022). Findings from a two-wave study indicate that SI is positively associated with perceived task performance (Liaquat & Escartín, 2025). Studies emphasize the need to study SI over time (Hamalainen et al., 2018; Jumisko-Pyykko et al., 2022) to understand the variability or changes in SI. Systems intelligence posits that factors such as systemic perception, attitude, systemic thinking, and action are key determinants of success, and successful actions. The present study proposes that perceived SI positively affects self-ratings of job performance over time. Actively regulating and optimizing resources can lead to better outcomes in terms of motivation and overall job performance. We test this hypothesis using a cross-lagged design across three waves:

**H1.**  *SI at Time 1 will predict job performance at Time 2 and Time 3.*

#### 1.1.2. Systems Intelligence and Job Crafting

Job crafting acts as a work booster for employees, enhancing their sense of control, leading to improved well-being and job performance (Tims et al., 2013, 2015). Job crafting is associated with various personal characteristics, such as personality (Bakker et al., 2012) and temperament (Gordon et al., 2015). Moreover, personal resources, which are an antecedent to job crafting, exhibit a positive correlation with work outcomes such as engagement (Van Wingerden et al., 2017). Notably, personal resources and job resources have been positively related (Bakker & Demerouti, 2014; Xanthopoulou et al., 2007, 2009a). A cross-sectional study concluded that personal resources (self-efficacy and resilience) positively affect employees’ perceptions of opportunities for crafting their jobs (Van Wingerden & Poell, 2019). To the best of our knowledge, no previous study has explored the association between SI and job crafting. We argue that SI will be related to job crafting behaviours for the following reasons. First, previous studies have argued that personal resources play a key role in job resources like crafting behaviours at work (Xanthopoulou et al., 2007; Bakker & Demerouti, 2014; Miraglia et al., 2017; D. Robledo et al., 2021). This is due to the fact that personal resources refer to the psychological traits or qualities of a person that are linked with resilience, thus showing the capability to manage and shape one’s environment effectively (Xanthopoulou et al., 2007). Second, as SI captures adaptation and adjustment in the environment including competencies such as positive engagement, positive attitude, effective responsiveness, and wise action (Hämäläinen & Saarinen, 2006), these competences are conducive to engaging in job crafting. Job crafting entails employees taking proactive and self-initiated steps to modify their job tasks, in order to achieve a better fit between their personal strengths, job demands and work outcomes (Wrzesniewski & Dutton, 2001). Therefore, SI should facilitate the proactivity needed to job craft. Third, personal resources can serve as antecedents of job resources or facilitate resources, a high level of personal resources can boost existing job resources (Galanakis & Tsitouri, 2022). For instance, individuals with strong personal resources (such as SI) may possess a proactive mindset, enabling them to engage in activities like job crafting. In light of the above arguments, we hypothesize:

**H2.**  *SI at Time 1 will predict job crafting at Time 2 and Time 3.*

#### 1.1.3. Systems Intelligence, Job Crafting and Job Performance

JD-R has been widely applied in job crafting (JC) research. JC recognised as a mediator in the JD-R model, which focuses on the interplay between job demands and job resources as well as their impact on employee well-being and performance (Bakker & Demerouti, 2014). Theorizing JD-R, job crafting may explain the relationship between systems intelligence and job performance.

So far, previous empirical findings have confirmed that job crafting mediates the positive impact of self-efficacy, proactive personality on career growth and employees’ performance outcomes (Miraglia et al., 2017; Rudolph et al., 2017; Miao et al., 2023). Positive psychological capital directly improves work engagement, with job crafting acting as a significant mediator (Park & Ha, 2025). Empirically, a longitudinal structural equation model confirmed the mediational role of job crafting in the association between psychological capital and job satisfaction, controlling for gender, education, age and job tenure (Cenciotti et al., 2017). Findings of time-lagged research, following the JD-R model, confirmed that job crafting at T2 mediates the association between work engagement (T1) and T3 job performance and flourishing (E. Robledo et al., 2019). The literature shows that personal resources (self-efficacy, proactive personality) and work outcomes are mediated by crafting behaviours. Job crafting allows employees to craft tasks according to their needs and desires.

**Figure 1:**
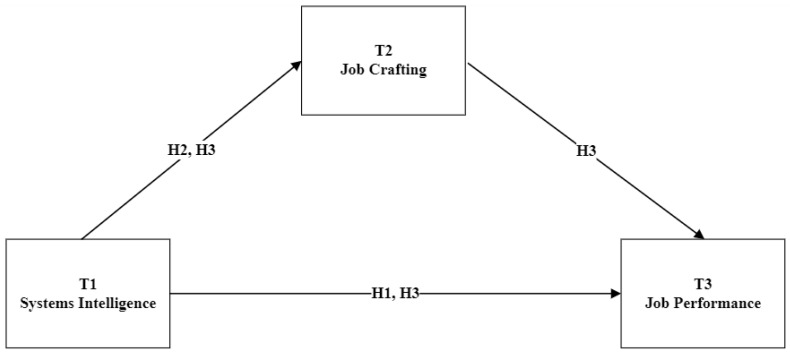
Research Model.

Employees who have high systems intelligence may use their cognitive abilities to create more engaging tasks to enhance their performance for the following reasons. First, employees with higher SI have a better awareness of systems and the environment, allowing them to adjust their tasks to optimise performance (Törmänen et al., 2021; Hamalainen et al., 2018). Second, the ability to craft one’s job according to individual preferences and strengths is an essential pathway through which SI can influence job performance. Employees with high SI levels can improve overall performance by using their cognitive abilities to modify and redesign their work conditions (Jumisko-Pyykko et al., 2022; Liaquat & Escartín, 2025). Therefore, we hypothesize that job crafting will mediate the association between SI and job performance (see Figure 1):

**H3.**  *Job crafting at Time 2 mediates the relationship between SI at Time 1 and job performance at Time 3.*

## 2. Method

We adopted a three-wave longitudinal design to study our variables over time. This approach enables causal inferences by capturing changes and establishing time-based sequences (M. Wang et al., 2017).

### 2.1. Sample

Participants were recruited from multinational organisations (banks, telecommunications, information technology, industries and engineering) in Pakistan. We selected different organizations and administered an online survey across three lags. We contacted the Human Resources (HR) departments of different organisations and administered an online survey across three time points. Only employees with a university education were recruited. Participation was voluntary. Informed consent was obtained from participants after they logged in to the online survey. In wave one, 380 participants responded to the online survey and 303 completed the survey. In wave two, 212 participants completed the survey, of whom 131 (62%) were male and 81 (38% were female). In wave three, 99 participants, 51 male employees (51%) and 48 female employees (49%), completed the survey. Therefore, the final sample comprised 99 employees who had completed the surveys at three different time points (response rate: 33%). Of these, 52% of the participants were aged between 20 and 30, 41% were under 40, 4% were under 50, and 3% were above 50 (see Table 1 for detailed demographic information).

We calculated the group differences in the measurements scores (all variables of the study) between the respondents who had completed all the surveys (T1, T2 and T3) and the respondents who had completed only the T1 survey. A series of T-tests were analysed, based on the SI scores (t = 2432, *p* = 0.758), job crafting (t = 2.014, *p* = 0.821), job performance (t = 0.714, *p* = 0.272), age (t = 0.104, *p* = 0.748) and gender (t = 2.126, *p* = 0.004). To control the selection bias on account of respondents who had dropped out, we assessed whether the dropout group (N = 204) differed from the participants who completed all three surveys (N = 99) in terms of age, gender and the study variables. Table 2-a indicates that the sample differed in terms of gender, but no significant differences were found in the scores of the study variables. We conducted two chi-square tests to determine the distribution of participants (see Table 2-b). We found no significant age and gender differences between the group that completed all three surveys and the drop out group. Therefore, we do not assume selection bias, as the total sample remained comparable to the overall study population.

### 2.2. Measures

All measures included in the study were in English as participants had adequate education levels to be proficient in English language (Haider et al., 2020). Cronbach’s alpha for our study variables is presented in Table 3.

#### 2.2.1. Systems Intelligence Inventory

The self-reported systems intelligence inventory (Törmänen et al., 2016) consists of 32 items with 7-point Likert scale ranging from 0 = never to 6 = always. Sample items include: I get a sense of what is essential to a given situation, I keep my cool even when situations are not under control, I think about the consequences of my actions, and I have a positive outlook on the future. Satisfactory reliability α = 0.94 was reported for SI inventory (Liaquat & Escartín, 2025).

#### 2.2.2. Perceived Job Performance

The Individual Work Performance Questionnaire (IWPQ) is a self-report (Koopmans et al., 2012) scale containing 18 items using a 5-point Likert scale (from 0 = seldom to 4 = always). It measures three dimensions: task performance (items 1 to 5), contextual performance (items 6 to 13) and counterproductive work behaviours (items 14 to 18). Sample items are as follows: I managed my time well; I worked on keeping my work skills up to date; and I talked to colleagues about the negative aspects of my work. Internal consistency ranges from α = 0.79 to α = 0.89 (Koopmans et al., 2016) and α = 0.72 to α = 0.84 (Akram & Siddiqui, 2019).

#### 2.2.3. Job Crafting

The job crafting scale (Sekiguchi et al., 2017) measures the original concept of crafting at work (Wrzesniewski & Dutton, 2001). The scale has nine items and uses a 7-point Likert scale (from strongly disagree = 1 to agree = 7 strongly). It measures three dimensions: task crafting, rational crafting and cognitive crafting. The sum score of all dimensions depicts the final job crafting score. Sample items from each dimension are as follows: I change the content and/or procedure of my job to be more desirable; I actively interact with people through my job; and I reframe my job as significant and meaningful, respectively. This measure has significant internal consistency; Cronbach’s reliability was 0.80 in study 1 with part-time employees and 0.90 in study 2 with full-time employees (Sekiguchi et al., 2017).

### 2.3. Procedure

Data collection was carried out after obtaining approval from the ethical committee of the University of the first author. We collected the data with the help of the HR departments of the companies in Pakistan. Participants were informed about the research, and an online survey link was shared via email. Participation was voluntary, and the confidentiality of the data was ensured. Informed consent was provided electronically before the survey. Responses to the questionnaires were matched with the emails provided by participants during each of the three waves. We collected data across three waves with two-months lag between each: the first wave from April to May 2021, the second wave from June to July 2021 and the third wave in from August to September 2021. Initially, 380 participants responded via the online survey at time 1. We excluded respondents who had just entered their email without consent (n = 20), deleted double responses (n = 30) and removed participants who only consented without any response (n = 17). A total of 303 completed responses were left. The same participants were approached again for T2 and T3. At second wave, we received a total of 212 participants after removing participants without consent (n = 8), deleting double responses (n = 10) and excluding participants who had only consented without any answer (n = 20). At time 3, we initially collected 100 responses, and after data cleaning, we retained a total of 99 participants. To minimise response bias and retain all information for the model, we employed the Full Information Maximum Likelihood (FIML) method with missing values (Duncan et al., 2006).

### 2.4. Data Analysis

We tested the reliability metrics of the measurement, the correlation for each variable for each lag and the longitudinal meditation with three-wave data. We estimated a three waves autoregressive cross-lagged model to test the indirect effect of paths: (a) SI T1 to job crafting T2, (b) job crafting T2 to job performance T3. Using a small sample size, we employed observed variables by reducing free estimated parameters (Avanzi et al., 2021; Balducci et al., 2020; Xanthopoulou et al., 2009a). Data were analysed using SPSS 29 (Statistical Package for Social Science) and Mplus 8 (Muthén & Muthén, 2017).

The model fit was evaluated by chi-square (χ^2^) statistic, Comparative Fit Index (CFI), Tucker–Lewis Index (TLI), Standardised Root Mean Square Residual (SRMR), and Root Mean Square Error of Approximation (RMSEA). Acceptable values for CFI and TLI range from 0 to 1, and hence, the higher the value, the better the fit. A value of 0.95 or higher indicates an acceptable model fit with a sample of 500 and more than 1000 participants. Values below 0.9 are also considered a fair fit of the model for a sample size of less than 250 or equal. For SRMR and RMSEA, a value of 0 indicates model fit, whereas values of 0.08, 0.05 or lower indicate a good fit to the data (Bentler, 1990; Hu & Bentler, 1999).

We specified a cross-lagged panel model with three latent variables across three time points. We performed simulation analysis to analyse the statistical power and investigate the hypothesised mediation of SI at T1 to job crafting T2 and job crafting at T2 to job performance T3. Autoregressive paths were specified to be 0.50, whereas cross-lagged paths from T1 SI to T2 job crafting and T2 job crafting to T3 job performance were found to be at 0.25 (Cohen, 1992). This test was performed using pwrSem (Y. A. Wang & Rhemtulla, 2021). With 1000 simulations, n = 99 and alpha value = 0.05, the power of T1 SI to T2 job crafting was 0.86, whereas the power of T2 job crafting to T3 job performance was 0.86, above the threshold of 0.80 (Boomsma, 2013; Muthén & Muthén, 2002; Wolf et al., 2013). It reflects a low to medium effect size (Avanzi et al., 2021; Qin, 2024). Please refer to Supplementary Materials for details.

#### Common Method Bias Test

To examine potential common method bias, we conducted Harman’s single-factor test following Podsakoff et al. (2003). All measurement items were entered into an exploratory factor analysis (EFA) using unrotated principal component analysis. The first factor accounted for 23.1% of the total variance, well below the 50% threshold, and multiple factors with eigenvalues greater than 1 emerged. These results indicate that common method bias was not a significant concern in this study.

## 3. Results

Descriptive statistics are shown in Table 3. All the measures showed significant internal consistency (Cronbach, 1951). The reliability estimates of SI, job crafting and job performance were found to be above the acceptable value of 0.70 (Hair, 2010).

**Figure 2:**
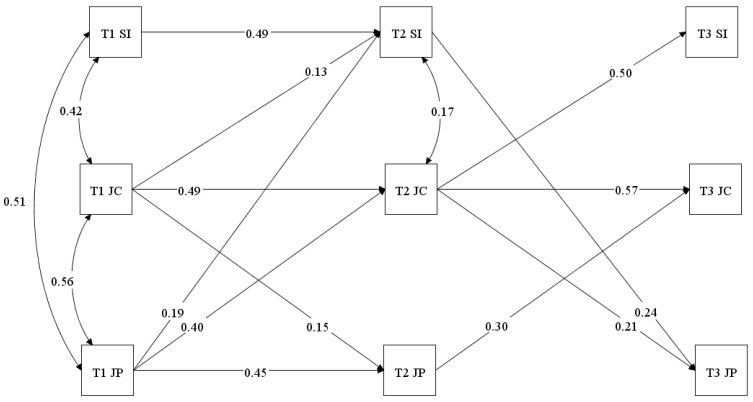
Significant cross-lagged paths from T1 to T2 and from T2 to T3 of systems intelligence, job crafting and job performance.

Correlational matrices suggest that higher T1 SI is associated with increased job crafting and job performance at different time points (T1, T2, and T3). Additionally, job crafting at T1 positively correlate SI at T2 and job crafting at T2 as well as job performance at T3. To test our hypotheses, we analysed a cross-lagged path model (without any constraint) using SI, job crafting and job performance. The baseline model statistics are shown in Table 4. All significant paths are shown in Figure 2.

In this model, we tested the paths from T1 to T2 and T2 to T3 of SI, job crafting and performance. Values of model fit were as follows: X^2^ = 56.538, df = 8, CFI = 0.951, TLI = 0.981, RMSEA = 0.042 and SRMR = 0.030. Hypothesis 1 indicated that SI would predict job performance over time. SI at time1 predicted job performance at T3 (β = 0.17 *), but not at T2. However, T2 SI predicted job performance at T3 (β = 0.24 **). To sum up, hypothesis 1 was partially supported.

Hypothesis 2 indicated that SI would predict job crafting over time. SI at time 1 was not significantly related to job crafting at time 2 (β = −0.22) nor at time 3 (β = −0.09). Further, SI at time 2 was not significantly related to job crafting at T3 (β = 0.005, *p* = 0.91). Therefore, hypothesis 2 was not supported. The third and final hypothesis indicated a mediation path from SI to job performance through job crafting. We also tested the indirect effect of job crafting as a mediator between SI and job performance. No mediation was found yielding no support for Hypothesis 3.

## 4. Discussion

The purpose of the present study was to explore the relationship between systems intelligence, job crafting and job performance over time. The findings revealed that SI at Time 1 was a strong predictor of job performance at Time 3, but not at Time 2. While SI at Time 2 significantly predicted performance at Time 3. SI showed no significant predictive relationship with job crafting over time. Similarly, we found that job crafting did not mediate the relationship between SI and job performance. These results indicate that systems intelligence and job crafting have independent effects on job performance. The effect of systems intelligence on job performance is aligned with the prior empirical evidence (Jumisko-Pyykko et al., 2022; Sasaki, 2017; Törmänen et al., 2021; Liaquat & Escartín, 2025), which highlight its role in equipping individuals with the ability to navigate complex systems and systemic thinking. This capacity allows individuals to effectively respond to and manage workplace challenges.

An unexpected finding is that systems intelligence does not predict job crafting. Several potential explanations may explain this. First, it might be the type of personal resources (Xanthopoulou et al., 2007). For instance, employees with higher SI may have an affective cognitive level and skills to enhance job performance in ways beyond job crafting. Another possibility is that high SI individuals may feel their existing personal resources sufficiently align with their strengths. This suggests that even though individuals with higher SI might be better equipped to manage and improve their work environment, they might not necessarily engage in job-crafting behaviours.

Findings indicated that job crafting did not act as a mediator in the relationship between SI and JP over time. These results indicate that the association between SI and job performance does not appear to be influenced or explained by the extent to which employees proactively modify their job tasks and roles to better align with their personal strengths and preferences, as measured by job crafting activities. This finding is not aligned with previous studies that established job crafting as a key mediating mechanism linking positive psychological capital with improved work engagement (Park & Ha, 2025), high-performance work systems with career growth (Miao et al., 2023), self-efficacy with job performance (Miraglia et al., 2017), and individual differences and job characteristics with engagement, performance, and well-being (Rudolph et al., 2017; E. Robledo et al., 2019). Our findings do not support the idea that individuals with high level of systems intelligence engage in job crafting behaviours that subsequently lead to improvements in job performance over time. This may be because key SI factors, such as systemic thinking and effective responsiveness, contribute directly to successful outcomes (Törmänen et al., 2021). SI enables individuals to adapt to complex environments and optimize work processes without the need for deliberate job crafting. Thus, SI may influence job performance through alternative mechanisms, such as strategic decision-making, problem-solving, or adaptive behaviours, rather than through job crafting. Other job resources such as social support, autonomy, organizational justice, (Tang et al., 2024; Deng et al., 2021; Mazzetti et al., 2023) and happiness at work (Fröhlich et al., 2025) might mediate the relationship between personal resources (such as SI) and job performance. Additionally, our findings are aligned with those of E. Robledo et al. (2019) in that job crafting behaviours seem to have a cumulative effect on job performance over time. One explanation for this might be that employees who craft on a regular basis are continually aligning their tasks with their strengths and values. This continual relevance of job resources fosters sustained engagement, motivation, and gradual improvements in performance over time. Furthermore, as employees modify their tasks through job crafting, they likely develop skills and personal resources that make them effective and resilient, facilitating consistent job performance.

### 4.1. Theoretical Implications

This study thus contributes to the literature on the JD-R model by highlighting the distinct contribution of personal resources in predicting job performance over time. This study identified SI as a distinct personal resource. Unlike traditional psychological resources such as optimism (Xanthopoulou et al., 2009a) and resilience (Van Wingerden & Poell, 2019), SI reflects a higher-order capability that integrates cognitive, emotional, and systemic thinking, enabling employees to align personal strengths in complex work contexts. Our findings support the theory of resource substitution (Demerouti & Bakker, 2023), which suggests that a resource has a stronger impact when alternative resources are limited, whereas the presence of multiple resources reduces the effectiveness of each as they compensate for one another in shaping job outcomes. In this context, strong personal resources may lessen employees’ dependence on job resources for job performance, indicating that those employees with well-developed personal resources are less reliant on external job resources to achieve positive work outcomes. Individuals with high systems intelligence (SI) may already possess cognitive and behavioural strategies that enhance job performance, diminishing their need for job crafting as an intermediary mechanism. Thus, SI may function as a substitute for job crafting, potentially explaining the absence of the expected mediating effect. Employees with high SI may demonstrate adaptability and adjustment at their workplace including competencies such as, positive engagement, positive attitude, effective responsiveness, and wise action (Hämäläinen & Saarinen, 2006). SI has multi-dimensional competencies (Törmänen et al., 2016), which can mitigate the need for traditional job resources like job crafting. Employees can navigate the challenges more efficiently and successfully by leveraging their personal strengths, skills, and competencies.

### 4.2. Practical Implications

Organizations can benefit by identifying and developing employees with higher levels of systems intelligence (SI) through targeted training programs, mentorship, coaching, and job design that encourages systemic thinking, adaptability, and problem-solving. SI is particularly relevant in today’s interconnected and dynamic work environments, as it promotes a deeper understanding of complex systems and fosters effective responses to challenges. Unlike traditional ‘top-down’ approaches, SI fosters a people-centered organizational culture through a ‘bottom-up’ approach that empowers individuals and teams (Törmänen et al., 2021).

At both the individual and organizational levels, SI contributes to success by fostering psychological empowerment, positive engagement, and proactive workplace behaviours. Leaders can utilize SI to identify opportunities, recognize workplace strains, and prevent potential challenges (Hamalainen et al., 2018; Sasaki, 2017). Organizations can foster SI among employees by implementing group assessments, targeted training programs, and structured interventions aimed at developing systemic thinking, wise action, and effective responsiveness.

At the individual level, employees can enhance their SI capabilities through training, self-development tools, and mindset shifts that emphasize positive attitudes, systemic thinking, and proactive problem-solving. Encouraging these behaviours enables individuals to navigate complexity more effectively and contribute to organizational resilience and innovation. By integrating SI-focused strategies across different levels, businesses can build a more adaptive, resilient, and growth-oriented work environment that supports both personal, organizational and collective success.

### 4.3. Limitations and Future Research

The limitations of the present study are worthy of mention. We chose the 2-month time lag between three waves of data for the purpose of convenience, and this may not have been an adequate time lapse for mediation to be observed. Avanzi et al. (2021) suggested that a year is a reasonable time to ascertain the mediational aspects. SI and job crafting had a positive correlation, but no significant association was found over time. These findings may be due to the time lag and small sample size. In that sense, another limitation was our small longitudinal sample, a standard limitation in longitudinal designs (Avanzi et al., 2021). The response rates for our study were relatively low: 69% at T2 (212 out of 303 respondents) and 32% at T3 (99 out of 303 respondents). Our sample included minor gender differences and several professionals with varying levels of autonomy, less decision-making, and job complexity. The nature of jobs and the type of organisation might affect crafting behaviour and job performance (Miraglia et al., 2017). Self-reported questionnaires can cause common method variance. Data were collected during the COVID-19 pandemic when remote working and working from home were in place. Therefore, the work situation and job resources experienced by participants may have differed to their pre-COVID-19 working conditions.

Future research should focus on collecting objective performance measures, such as supervisor-rated job performance and co-worker evaluation of SI, to reduce reliance on self-reports and enhance validity. Future research should strive for more balanced gender representation or larger sample sizes to robustly examine gender effects without inflating their importance. Additionally, job demands, particularly job stressors, could serve as potential mediators in the relationship between SI and performance outcomes, warranting further exploration through longitudinal studies with extended time lags to capture delayed mediational effects. Building on recent findings, scholars should identify theoretically grounded and context-specific moderators, such as flexible work arrangements and role ambiguity (L. Wang & Xie, 2023), which influence SI across diverse settings. In particular, within the Pakistani work-context, characterized by rigid hierarchies and limited work–life balance, the role of job and personal resources in supporting employee mental health and engagement is especially pertinent (Fazal et al., 2022).

Moreover, future studies could conceptualize SI as a form of playful work design, investigating how it complements existing job crafting strategies in enhancing employees’ momentary experiences of autonomy, relatedness, and competence. Such integration could significantly advance theory on proactive work behaviour and self-determined motivation (Scharp et al., 2022). Future research might take our theorizing further by exploring how systems intelligence and job crafting interact with job demands. This would add more depth to the boundary conditions of the resource substitution perspective. To develop a more comprehensive understanding of SI and performance-related outcomes, researchers are encouraged to adopt a multi-level approach, examining individual, team, and organizational-level predictors. Finally, diary studies and mixed-method research designs can provide rich insights into the temporal dynamics and contextual nuances of systems intelligence, job crafting, performance, and work-related well-being, enabling stronger causal inferences.

## 5. Conclusions

This study has contributed towards tracing the associations among personal resources (SI), job resources (job crafting) and job performance using the JD-R model. Employing a three-wave longitudinal design, the study found no statistically significant mediation path between SI and job performance. In particular, SI at time 1 was found to positively affect job performance at time 3. Employees with high SI were more likely to demonstrate high job performance. The findings also demonstrated that the JD-R theory is a valuable framework for understanding SI (as a personal resources) and job performance. Therefore, SI may be seen as a feasible mechanism that underlies the positive relationship with job performance. This study has thus shed light on possible interventions related to SI, which can enhance performance, understanding of organisational systems, task requirements, positive engagement and effective responsiveness.

## Figures and Tables

**Table 1:** Descriptive Overview of Participant Demographics.

	Age Groups	N	%	Gender	N	%
T1	20–30	169	56	Male	182	60%
	30–40	108	36	Female	121	40%
	40–50	19	6			
	50–60	7	2			
	Total	303	100		303	
T2	20–30	114	54	Male	131	62%
	30–40	81	37	Female	81	38%
	40–50	11	6			
	50–60	6	3			
	Total	212	100		212	
T3	20–30	51	52	Male	51	51%
	30–40	41	41	Female	48	49%
	40–50	4	4			
	50–60	3	3			
	Total	99	100		99	

**Table 2:** (**a**) T-Test for participation and drop out; (**b**) Chi-Square Test.

(**a**)								
**Variables**		**N**	**M**	**SD**	**F**	**t**	**df**	***p***
Gender	Participation	99	1.43	0.498	4.819	1.284	301	0.029
	Drop out	204	1.36	0.481				
Age	Participation	99	1.59	0.714	0.104	0.587	301	0.748
	Drop out	204	1.53	0.718				
T1SI	Participation	99	128.43	25.27	0.095	2.432	301	0.758
	Drop out	204	120.41	27.67				
T1JC	Participation	99	47.95	9.48	0.051	2.014	301	0.821
	Drop out	204	45.74	8.74				
T1JP	Participation	99	51.54	10.89	1.212	0.714	301	0.272
	Drop out	204	50.51	12.11				
(**b**)								
**Variable**		**Participation**		**Dropout**	**N**	**χ2**	**df**	***p***
Age	20–30	51		118	169	3.349	3	0.341
	30–40	41		67	108			
	40–50	4		15	19			
	50–60	3		4	7			
Total		99		204	303			
Gender	Male	56		131	182	1.651	1	0.210
	Female	43		73	121			
Total		99		204	303			

**Table 3:** Descriptive statistics, reliability estimates, and Correlational matrices of three-wave measures.

	N	M	SD	Items	α	T1SI	T1JC	T1JP	T2SI	T2JC	T2JP	T3SI	T3JC	T3JP
T1SI	303	29.28	11.13	32	0.92	1	0.201 **	0.108	0.338 **	0.134	0.138 *	0.203 *	−0.004	0.194
T1JC	303	46.46	9.04	9	0.86		1	0.018	0.157 *	0.578 **	−0.012	0.169	0.076	0.208 *
T1JP	303	50.85	11.72	18	0.84			1	−0.020	0.012	0.475 **	−0.080	0.026	0.959 **
T2SI	212	31.37	11.50	32	0.94				1	0.326 **	0.052	0.853 **	−0.102	−0.046
T2JC	212	48.40	9.20	9	0.85					1	0.079	0.208 *	0.146	0.228 *
T2JP	212	53.39	10.81	18	0.80						1	−0.021	0.010	0.644 **
T3SI	99	30.46	11.66	32	0.92							1	−0.097	−0.065
T3JC	99	49.94	7.98	9	0.86								1	0.075
T3JP	99	51.74	11.09	18	0.81									1

Note: T1SI = time 1 systems Intelligence, T1JC = time 1 job crafting, T1JP = time 1 job performance, T2SI = time 2 systems Intelligence, T2JC = time 2 job crafting, T2JP = time 2 job performance, T3SI = time 3 systems Intelligence, T3JC = time 3 job crafting, T3JP = time 3 job performance, ** *p* < 0.01, * *p* < 0.05.

**Table 4:** Estimates and standardized direct effects of the model.

Directional Paths	Estimates	SE	*p*
T1SI → T1JC	0.42	0.047	0.000 ***
T1S1 → T1JP	0.51	0.042	0.000 ***
T1SI → T2JC	−0.02	0.043	0.61
T1SI → T2JP	0.06	0.055	0.30
T1SI → T3JC	0.09	0.050	0.07
T1SI → T3JP	0.17	0.067	0.01 *
T2SI → T2JC	0.17	0.056	0.002 **
T2SI → T2JP	0.07	0.057	0.22
T2SI → T3JC	0.00	0.044	0.91
T2SI → T3JP	0.24	0.059	0.000 ***
T3SI → T3JC	0.19	0.057	0.06
T3SI → T3JP	−0.06	0.057	0.25
T1JC → T1SI	0.42	0.047	0.000 ***
T1JC → T1JP	0.56	0.040	0.000 ***
T1JC → T2JC	0.49	0.042	0.000 ***
T1JC → T2SI	0.13	0.051	0.000 ***
T1JC → T2JP	0.15	0.056	0.008 **
T1JC → T3JC	0.11	0.054	0.03 *
T1JC → T3SI	−0.06	0.148	0.68
T1JC → T3JP	−0.18	0.072	0.01 *
T2JC → T2SI	0.17	0.056	0.000 ***
T2JC → T2JP	0.06	0.057	0.25
T2JC → T3JC	0.57	0.042	0.000 ***
T2JC → T3SI	0.50	0.052	0.000 ***
T2JC → T3JP	0.21	0.062	0.001 **
T3JC → T3SI	0.19	0.057	0.06
T3JC → T3JP	0.09	0.057	0.11

Note. T1SI = systems intelligence at time 1, T1JC = job crafting at time 1, T1JP = job performance at time 1, T2SI = systems intelligence at time 2, T2JC = job crafting at time 2, T2JP = job performance at time 2, T3SI = systems intelligence at time 3, T3JC = job crafting at time 3, T3JP = job performance at time 3, * *p* < 0.05, ** *p* < 0.01, *** *p* < 0.001.

## Data Availability

The data supporting the findings of this study are not publicly available due to participant confidentiality but can be obtained from the corresponding author upon reasonable request.

## Supplementary Materials

The following supporting information can be downloaded at: https://www.mdpi.com/article/10.3390/bs15091255/s1.

## References

[B1-behavsci-15-01255] Akram H., Siddiqui D. A. (2019). Impact of emotional intelligence on individual work performance of employees with the mediating role of decision-making styles: Evidence from Pakistan. SSRN Electronic Journal.

[B2-behavsci-15-01255] Avanzi L., Perinelli E., Bressan M., Balducci C., Lombardi L., Fraccaroli F., Van Dick R. (2021). The mediational effect of social support between organizational identification and employees’ health: A three-wave study on the social cure model. Anxiety, Stress, & Coping.

[B3-behavsci-15-01255] Bakker A. B., Demerouti E. (2007). The job demands-resources model: State of the art. Journal of Managerial Psychology.

[B4-behavsci-15-01255] Bakker A. B., Demerouti E., Cooper C. L. (2014). Job demands-resources theory. Wellbeing.

[B5-behavsci-15-01255] Bakker A. B., Demerouti E. (2017). Job demands–resources theory: Taking stock and looking forward. Journal of Occupational Health Psychology.

[B6-behavsci-15-01255] Bakker A. B., Tims M., Derks D. (2012). Proactive personality and job performance: The role of job crafting and work engagement. Human Relations.

[B7-behavsci-15-01255] Balducci C., Baillien E., Broeck A. V. D., Toderi S., Fraccaroli F. (2020). Job demand, job control, and impaired mental health in the experience of workplace bullying behavior: A two-wave study. International Journal of Environmental Research and Public Health.

[B8-behavsci-15-01255] Bentler P. M. (1990). Comparative fit indexes in structural models. Psychological Bulletin.

[B9-behavsci-15-01255] Boomsma A. (2013). Reporting monte Carlo studies in structural equation modeling. Structural Equation Modeling: A Multidisciplinary Journal.

[B10-behavsci-15-01255] Cenciotti R., Alessandri G., Borgogni L. (2017). Psychological capital and career success over time: The mediating role of job crafting. Journal of Leadership & Organizational Studies.

[B11-behavsci-15-01255] Cohen J. (1992). Statistical power analysis. Current Directions in Psychological Science.

[B12-behavsci-15-01255] Cronbach L. J. (1951). Coefficient alpha and the internal structure of tests. Psychometrika.

[B13-behavsci-15-01255] Demerouti E., Bakker A. B. (2023). Job demands-resources theory in times of crises: New propositions. Organizational Psychology Review.

[B14-behavsci-15-01255] Demerouti E., Bakker A. B., Gevers J. M. P. (2015). Job crafting and extra-role behavior: The role of work engagement and flourishing. Journal of Vocational Behavior.

[B15-behavsci-15-01255] Demerouti E., Bakker A. B., Nachreiner F., Schaufeli W. B. (2001). The job demands-resources model of burnout. The Journal of Applied Psychology.

[B16-behavsci-15-01255] Deng J., Liu J., Guo Y., Gao Y., Wu Z., Yang T. (2021). How does social support affect public service motivation of healthcare workers in China: The mediating effect of job stress. BMC Public Health.

[B17-behavsci-15-01255] Drigas A., Papoutsi C., Skianis C. (2023). Being an emotionally intelligent leader through the nine-layer model of emotional intelligence—The supporting role of new technologies. Sustainability.

[B18-behavsci-15-01255] Duncan S. C., Duncan T. E., Strycker L. A. (2006). Alcohol use from ages 9 to 16: A cohort-sequential latent growth model. Drug and Alcohol Dependence.

[B19-behavsci-15-01255] Fazal S., Masood S., Nazir F., Majoka M. I. (2022). Individual and organizational strategies for promoting work–life balance for sustainable workforce: A systematic literature review from Pakistan. Sustainability.

[B20-behavsci-15-01255] Fröhlich P., Radaca E., Diestel S. (2025). When happiness strengthens engagement and performance: The role of happiness at work as a resource for experienced employees and newcomers. Frontiers in Psychology.

[B21-behavsci-15-01255] Galanakis M. D., Tsitouri E. (2022). Positive psychology in the working environment. Job demands-resources theory, work engagement and burnout: A systematic literature review. Frontiers in Psychology.

[B22-behavsci-15-01255] Gonzalez-Mulé E., Kim M. M., Ryu J. W. (2021). A meta-analytic test of multiplicative and additive models of job demands, resources, and stress. Journal of Applied Psychology.

[B23-behavsci-15-01255] Gordon H. J., Demerouti E., Le Blanc P. M., Bipp T. (2015). Job crafting and performance of Dutch and American health care professionals. Journal of Personnel Psychology.

[B24-behavsci-15-01255] Haider S., Fatima N., Pablos-Heredero C. D. (2020). A three-wave longitudinal study of moderated mediation between perceptions of politics and employee turnover intentions: The role of job anxiety and political skills. Revista de Psicología Del Trabajo y de Las Organizaciones.

[B25-behavsci-15-01255] Hair J. F. (2010). Multivariate data analysis: A global perspective.

[B27-behavsci-15-01255] Hämäläinen R. P., Saarinen E. (2006). Systems intelligence: A key competence in human action and organisational life. Reflections: The SoL Journal.

[B26-behavsci-15-01255] Hamalainen R. P., Saarinen E., Tormanen J. (2018). Systems intelligence: A core competence for next-generation engineers?. 2018 IEEE International Conference on Teaching, Assessment, and Learning for Engineering (TALE).

[B28-behavsci-15-01255] Hobfoll S. E., Halbesleben J., Neveu J.-P., Westman M. (2018). Conservation of resources in the organizational context: The reality of resources and their consequences. Annual Review of Organizational Psychology and Organizational Behavior.

[B29-behavsci-15-01255] Hobfoll S. E., Johnson R. J., Ennis N., Jackson A. P. (2003). ‘Resource loss, resource gain, and emotional outcomes among inner city women’: Correction to Hobfoll et al. (2003). Journal *of Personality and Social Psychology*.

[B30-behavsci-15-01255] Hu L., Bentler P. M. (1999). Cutoff criteria for fit indexes in covariance structure analysis: Conventional criteria versus new alternatives. Structural Equation Modeling: A Multidisciplinary Journal.

[B31-behavsci-15-01255] Jumisko-Pyykko S., Törmänen J., Vanni K., Hämäläinen R., Saarinen E., Salminen V. (2022). Systems intelligence, perceived performance and wellbeing. Human factors, business management and society.

[B32-behavsci-15-01255] Koltai J., Schieman S. (2015). Job pressure and SES-contingent buffering: Resource reinforcement, substitution, or the stress of higher status?. Journal of Health and Social Behavior.

[B33-behavsci-15-01255] Koopmans L., Bernaards C., Hildebrandt V., Van Buuren S., Van Der Beek A. J., De Vet H. C. W. (2012). Development of an individual work performance questionnaire. International Journal of Productivity and Performance Management.

[B34-behavsci-15-01255] Koopmans L., Bernaards C. M., Hildebrandt V. H., Lerner D., De Vet H. C. W., Van Der Beek A. J. (2016). Cross-cultural adaptation of the individual work performance questionnaire. Work.

[B35-behavsci-15-01255] Liaquat S., Escartín J. (2025). Systems intelligence and job autonomy in managing stressors and performance: A time-lagged study in multinational firms. Sustainability.

[B36-behavsci-15-01255] Lu C., Wang H., Lu J., Du D., Bakker A. B. (2014). Does work engagement increase person–job fit? The role of job crafting and job insecurity. Journal of Vocational Behavior.

[B37-behavsci-15-01255] Mazzetti G., Robledo E., Vignoli M., Topa G., Guglielmi D., Schaufeli W. B. (2023). Work engagement: A meta-analysis using the job demands-resources model. Psychological Reports.

[B38-behavsci-15-01255] Miao R., Yu J., Bozionelos N., Bozionelos G. (2023). Organizational career growth and high-performance work systems: The roles of Job Crafting and organizational innovation climate. Journal of Vocational Behavior.

[B39-behavsci-15-01255] Miraglia M., Cenciotti R., Alessandri G., Borgogni L. (2017). Translating self-efficacy in job performance over time: The role of job crafting. Human Performance.

[B40-behavsci-15-01255] Muthén L. K., Muthén B. (2017). Mplus user’s guide.

[B41-behavsci-15-01255] Muthén L. K., Muthén B. O. (2002). How to use a Monte Carlo study to decide on sample size and determine power. Structural Equation Modeling.

[B42-behavsci-15-01255] Neuber L., Englitz C., Schulte N., Forthmann B., Holling H. (2021). How work engagement relates to performance and absenteeism: A meta-analysis. European Journal of Work and Organizational Psychology.

[B43-behavsci-15-01255] Park S., Ha Y. (2025). The relationship between positive psychological capital and work engagement in clinical nurses: Mediation effect of job crafting. BMC Nursing.

[B44-behavsci-15-01255] Podsakoff P. M., MacKenzie S. B., Lee J.-Y., Podsakoff N. P. (2003). Common method biases in behavioral research: A critical review of the literature and recommended remedies. Journal of Applied Psychology.

[B45-behavsci-15-01255] Qin X. (2024). Sample size and power calculations for causal mediation analysis: A tutorial and Shiny app. Behavior Research Methods.

[B46-behavsci-15-01255] Robledo D., Vázquez-Delfín E., Freile-Pelegrín Y., Vásquez-Elizondo R. M., Qui-Minet Z. N., Salazar-Garibay A. (2021). Challenges and opportunities in relation to sargassum events along the Caribbean Sea. Frontiers in Marine Science.

[B47-behavsci-15-01255] Robledo E., Zappalà S., Topa G. (2019). Job crafting as a mediator between work engagement and wellbeing outcomes: A time-lagged study. International Journal of Environmental Research and Public Health.

[B48-behavsci-15-01255] Rofcanin Y., Bakker A. B., Berber A., Gölgeci I., Las Heras M. (2019). Relational job crafting: Exploring the role of employee motives with a weekly diary study. Human Relations.

[B49-behavsci-15-01255] Ross C. E., Mirowsky J. (2010). Gender and the health benefits of education. The Sociological Quarterly.

[B50-behavsci-15-01255] Rudolph C. W., Katz I. M., Lavigne K. N., Zacher H. (2017). Job crafting: A meta-analysis of relationships with individual differences, job characteristics, and work outcomes. Journal of Vocational Behavior.

[B51-behavsci-15-01255] Saarinen E., Hämäläinen R. P. (2004). Systems intelligence: Connecting engineering thinking with human sensitivity. Systems intelligence—Discovering a hidden competence in human action and organizational life.

[B52-behavsci-15-01255] Sasaki Y. (2017). A note on systems intelligence in knowledge management. The Learning Organization.

[B53-behavsci-15-01255] Scharp Y. S., Bakker A. B., Breevaart K. (2022). Playful work design and employee work engagement: A self-determination perspective. Journal of Vocational Behavior.

[B54-behavsci-15-01255] Schaufeli W. B., Taris T. W., Bauer G. F., Hämmig O. (2014). A critical review of the job demands-resources model: Implications for Improving work and health. Bridging occupational, organizational and public health.

[B55-behavsci-15-01255] Sekiguchi T., Li J., Hosomi M. (2017). Predicting job crafting from the socially embedded perspective: The interactive effect of job autonomy, social skill, and employee status. The Journal of Applied Behavioral Science.

[B56-behavsci-15-01255] Senge P. M. (1990). The fifth discipline: The Art and Practice of the Learning Organization.

[B57-behavsci-15-01255] Tang H., An S., Zhang L., Xiao Y., Li X. (2024). The antecedents and outcomes of public service motivation: A meta-analysis using the job demands–resources model. Behavioral Sciences.

[B58-behavsci-15-01255] Tims M., Bakker A. B., Derks D. (2012). Development and validation of the job crafting scale. Journal of Vocational Behavior.

[B59-behavsci-15-01255] Tims M., Bakker A. B., Derks D. (2013). The impact of job crafting on job demands, job resources, and well-being. Journal of Occupational Health Psychology.

[B60-behavsci-15-01255] Tims M., Bakker A. B., Derks D. (2015). Job crafting and job performance: A longitudinal study. European Journal of Work and Organizational Psychology.

[B61-behavsci-15-01255] Törmänen J., Hämäläinen R. P., Saarinen E. (2016). Systems intelligence inventory. The Learning Organization.

[B62-behavsci-15-01255] Törmänen J., Hämäläinen R. P., Saarinen E. (2021). Perceived systems intelligence and performance in organizations. The Learning Organization.

[B63-behavsci-15-01255] Törmänen J., Hämäläinen R. P., Saarinen E. (2022). On the systems intelligence of a learning organization: Introducing a new measure. Human Resource Development Quarterly.

[B64-behavsci-15-01255] Van Wingerden J., Derks D., Bakker A. B. (2017). The impact of personal resources and job crafting interventions on work engagement and performance. Human Resource Management.

[B65-behavsci-15-01255] Van Wingerden J., Poell R. F. (2019). Antecedents of job crafting behavior within organizations: The role of personal resources, job resources and perceived opportunities to craft in employees proactive behavior. International Journal of Human Resource Studies.

[B66-behavsci-15-01255] Wang L., Xie T. (2023). Double-edged sword effect of flexible work arrangements on employee innovation performance: From the demands–resources–individual effects perspective. Sustainability.

[B67-behavsci-15-01255] Wang M., Beal D. J., Chan D., Newman D. A., Vancouver J. B., Vandenberg R. J. (2017). Longitudinal research: A panel discussion on conceptual issues, research design, and Statistical Techniques. Work, Aging and Retirement.

[B68-behavsci-15-01255] Wang Y. A., Rhemtulla M. (2021). Power analysis for parameter estimation in structural equation modeling: A discussion and tutorial. Advances in Methods and Practices in Psychological Science.

[B69-behavsci-15-01255] Wolf E. J., Harrington K. M., Clark S. L., Miller M. W. (2013). Sample size requirements for structural equation models: An evaluation of power, bias, and solution propriety. Educational and Psychological Measurement.

[B70-behavsci-15-01255] Wrzesniewski A., Dutton J. E. (2001). Crafting a job: Revisioning employees as active crafters of their work. The Academy of Management Review.

[B71-behavsci-15-01255] Xanthopoulou D., Bakker A. B., Demerouti E., Schaufeli W. B. (2007). The role of personal resources in the job demands-resources model. International Journal of Stress Management.

[B72-behavsci-15-01255] Xanthopoulou D., Bakker A. B., Demerouti E., Schaufeli W. B. (2009a). Reciprocal relationships between job resources, personal resources, and work engagement. Journal of Vocational Behavior.

[B73-behavsci-15-01255] Xanthopoulou D., Bakker A. B., Demerouti E., Schaufeli W. B. (2009b). Work engagement and financial returns: A diary study on the role of job and personal resources. Journal of Occupational and Organizational Psychology.

