# Supplementary material for: Working Differently, Performing Similarly: Systems Intelligence and Job Crafting as Predictors of Job Performance in a Three-Wave Longitudinal Study
Source: Behav Sci (Basel). 2025 Sep 14;15(9):1255. doi: 10.3390/bs15091255 (PMC12467773; doi:10.3390/bs15091255)
Supplement: Supplementary file 1 [file behavsci-15-01255-s001.zip › behavsci-3833916-supplementary.pdf]

Supplementary Materials

Power analysis

We did power analysis with online pwrSEM (Wang & Rhemtulla, 2021), available at https://yilinandrewang.shinyapps.io/pwrSEM/

A screenshot of the result and entered codes are below.

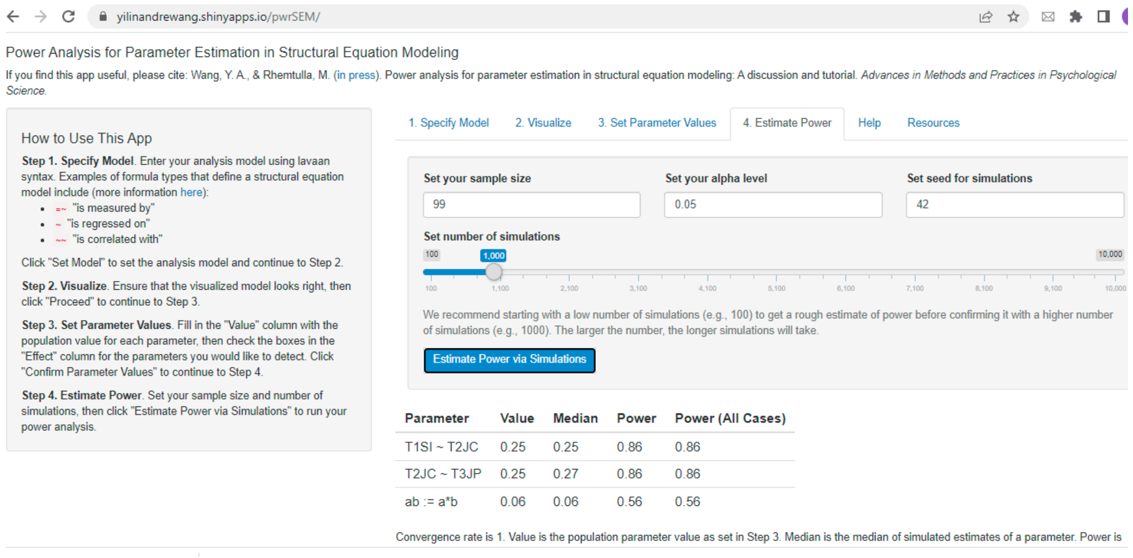

#Hypothesized Model

#Measurement models

T1JC =~ 1*T1JC_obs

T1SI =~ 1*T1SI_obs

T1JP =~ 1*T1JP_obs

T2JC =~ 1*T2JC_obs

T2SI =~ 1*T2SI_obs

T2JP =~ 1*T2JP_obs

T3JC =~ 1*T3JC_obs

T3SI =~ 1*T3SI_obs

T3JP T=~1*T3JP_obs

#Covariances

T1SI ~~ T1JC + T1JP

T1JC ~~ T1JP

T2SI ~~ T2JC + T3JP

T2JC ~~ T2JP

T3SI ~~ T3JC + T3JP

T3JC ~~ T3JP

#Autoregressive paths

T1SI ~ T2SI

T2SI ~ T3SI

T1JC ~ T2JC

T2JC ~ T3JC

```
T1JP ~ T2JP
T2JP ~ T3JP

#Cross-lagged paths
T1SI ~ a*T2JC + T2JP
#T1JC ~ T2SI #+ T2JP
#T1JP ~ T2JC + 21SI

#T2SI ~ T3JC + T3JP
T2JC ~ T3SI + b*T3JP
#T2JP ~ T3JC# + T3SI

#Indirect effect
ab := a*b
```
